# The Voluntary Hospitals in Danger; "Wake Up" Everybody!

**Published:** 1911-07-15

**Authors:** 


### The Hospital

A Journal of

The Medical Sciences and Hospital Administration.

New Series. No. 233, Vol. IX. [No. 1305, Vol. L.] SATURDAY, JULY 15, 1911

### The Voluntary Hospitals in Danger; "Wake Up" Everybody!

As we have already pointed out the voluntary hospital managers, both in London and throughout the country, have asked for a postponement of the Insurance Bill to give the necessary amount of time to enable it to be thoroughly examined, and its effects upon the voluntary hospital system thoroughly mastered. A Committee, as we stated last week, representing the voluntary hospitals, has asked for an interview with the Chancellor of the Exchequer, and we understand that the managers of the London hospitals through the Central Hospital Council have adopted a similar course. Up to the hour of our going to press no such interview had taken place or been arranged, so far as we could ascertain, and it seems to us that unless the managers of the hospitals bestir themselves their institutions are likely to suffer a mortal injury by the passing of this Insurance Bill, for in its present form it must tend to diminish the income of a great number of our most important voluntary hospitals to an extent which may render it difficult or impossible for them to continue the beneficent work upon which they are at present engaged. It is a very extraordinary thing that power should be taken in the Bill to provide large sums for the maintenance of institutions for the treatment of tuberculosis, while no provision is made to defray the cost of the hospital service which the State will require for insured persons under this Bill.

In our view the British Hospitals Association, the Central Hospital Council, and the managers of every important voluntary hospital throughout the country should assemble a great meeting in London and organise immediately a vigorous canvass of their influential supporters, whom they should get to write individually to the members of Parliament for their districts, pointing out that the voluntary hospital is threatened with destruction, and that the Bill cannot in the public interests be permitted to pass in its present form. We are anxious that this meeting of representatives of all the important hospitals throughout the country should be held in London within the next fortnight, because it is essential that the managers shall be in complete agreement with the policy which they intend unitedly to pursue in regard to this Insurance Bill. From time immemorial it has been the habit in this country for good people connected with voluntary hospitals to shudder whenever a suggestion has been made that the State shall contribute towards their support. In fact the State has all along contributed by subscriptions and grants from local authorities, municipalities, and poor-law guardians, and in the case of some cities like Manchester the municipality has been in the habit of giving substantial grants towards building funds, whenever a reconstruction or rebuilding scheme for their principal voluntary hospital has had to be carried out. There is nothing novel, therefore, in the idea that the State or local authority shall pay the voluntary hospitals for the treatment they give to State patients.

Recent legislation, including the Employers' Liability Act, and the new provision for school clinics in connection with day schools in large towns and communities, have proved in practice most unjust to the voluntary hospitals. This legislation has resulted in casting a great amount of increased work upon the voluntary hospitals, and in cases of accident, where an injured person has received large benefits under the Employers' Liability Act, the voluntary hospital where he may have been treated has had to bear all the cost of treatment without any repayment whatever. If the present Insurance Bill as it stands becomes law the State may force the voluntary hospitals practically to fill their beds with insured patients, and to undertake a great amount of additional work in every department without providing any means whereby the hospitals will be recouped by the State. The time has therefore come when the managers of every voluntary hospital must combine to insist upon just treatment by Parliament, seeing that modern legislation has tended greatly to add to the cost and work of the voluntary hospital, without providing funds to defray the cost of such work.

The clauses which affect voluntary hospitals are three, i.e. 8, 15, and 17. A few simple verbal amendments in the first two clauses and the deletion of clause 17 could put matters right and make provision for the payment *pro rata* by the State or local authority for the costs of any work which the passing of the Insurance Bill may cast upon the voluntary hospitals. Clause 17 should be deleted because it is quite certain that the managers of no well-managed voluntary hospital can accept subscriptions or donations from an approved society, for that would mean that for a relatively insignificant payment its members would consider themselves entitled to demand and have all the hospital care without further payment each year, which they might require. It is essential to protect the voluntary hospitals from the injury which has been done them by the recent legislation to which we have referred, and to find a way for their protection against any similar losses and difficulties owing to the passing of Mr. Lloyd George's Bill.

Some hospital managers seem to fear that a *pro rata* payment for the work actually required of the voluntary hospital by the State will injure the voluntary principle. On the contrary, it must in practice tend to strengthen the voluntary hospital by freeing it from many abuses and securing that only those patients who are properly entitled to free medical relief shall thenceforward receive it at one of these institutions. If the voluntary hospitals were to consent to accept doles or grants from the State—that is to say, a definite sum per annum without any regard to the work which they might do directly for the State—then their independnce would undoubtedly be affected adversely. A general subscription or payment by the State could not be accepted by any voluntary hospital without grave risk to the voluntary principle upon which their finances at present rest. Indeed, in our view, such a method of payment must ultimately end in the extinction of the voluntary hospitals altogether. On the other hand, if the managers of all the hospitals combine in defence of their rights at this important crisis in their history, and demand from the Government a *pro rata* payment for every insured patient for whom the State requires hospital care, the voluntary principle will be in no sense injured, because a rearrangement of the existing buildings and a strict classification of the patients will effectually prevent any such danger. It is one thing for a voluntary hospital to co-operate with the State by receiving its patients and treating them on the State's behalf at the State's cost. It is quite another thing to take a grant of money from the State for a voluntary hospital and apply it to the general purposes of the whole institution. We must preserve the voluntary principle of hospital management and hospital support in this country, and protect it with all our might. The present Bill as it stands threatens the voluntary hospitals, and it is essential that without one moment's avoidable delay voluntary hospital managers throughout the country should combine and, by organised effort, save their institutions from the financial dangers with which they are threatened at the moment by Mr. Lloyd George's Bill.

